# Medical Malpractice Lawsuits Involving Pediatric Trainees

**DOI:** 10.7759/cureus.42814

**Published:** 2023-08-01

**Authors:** Morgan T Bradley, Roei Golan, Vanessa Agudelo, Nicholas D Thomas, Katherine Donches

**Affiliations:** 1 College of Medicine, Florida State University College of Medicine, Tallahassee, USA; 2 Harvard Law School, Harvard University, Cambridge, USA; 3 Pediatrics, Children's Hospital of Philadelphia, University of Pennsylvania Perelman School of Medicine, Philadelphia, USA

**Keywords:** fellows, litigation, pediatrics, residents, lawsuits, malpractice

## Abstract

Introduction

Pediatric medical trainees, like other medical professionals, can be held accountable for their actions and may be included in malpractice lawsuits. The aim of this study was to investigate the sources of malpractice cases involving pediatric trainees in order to inform the development of strategies to protect against such incidents.

Methods

LexisNexis, an online public legal research database containing records from the United States, was retrospectively reviewed for malpractice cases involving pediatric interns, residents, or fellows from January 1, 2000, to December 31, 2021. Cases were included if malpractice occurred following the delivery of a newborn through the care of young adults up to age 21.

Results

A total of 56 cases were included, consisting of 10 pediatric interns, 43 second- or third-year residents, and 11 pediatric fellows as defendants. Seventeen cases (30.4%) led to patient mortality. Incorrect diagnosis or treatment was claimed in 45 cases (80.4%), delay in evaluation in 24 (42.9%), failure to supervise trainee in 22 (39.3%), trainee inexperience in 21 (37.5%), procedural error in 21 (37.5%), lack of informed consent of resident being involved in two (3.6%), prolonged operative time in one (1.8%), and lack of informed consent of procedure/complications in one (1.8%).

Conclusion

Malpractice cases involving pediatric trainees highlight the importance of adequate supervision by attending physicians. These concerns are not exclusive to interns and residents and necessitate action by all members of the healthcare team. Given the interplay of supervision and diagnostic accuracy, trainee education and faculty development should emphasize malpractice education and strategies to mitigate lawsuits to both improve patient outcomes and reduce the likelihood of future malpractice claims.

## Introduction

Despite decreasing rates of nationwide paid malpractice claims on behalf of physician trainees, significant uncertainty about how to protect oneself from being involved in such a lawsuit remains [1]. Substantial variation in the probability of involvement in a malpractice case exists across specialties; annually, 7.4% of physicians in the United States face a claim. Data for pediatricians show a 3.1% probability of facing a suit annually, classifying pediatrics as the second lowest-risk specialty for facing a suit, second only to psychiatry [2]. Pediatricians are estimated to have a 36% chance of facing their first claim by age 45, compared to 88% of physicians in high-risk specialties such as obstetrics and general surgery [2]. 

Trainees can be held accountable as defendants in lawsuits, and it is crucial to comprehend the circumstances surrounding these legal actions. By utilizing this understanding, trainees can be educated and future incidents can potentially be prevented. Approximately 10% of lawsuits against pediatricians occur during training [3]. Physician trainees are often assigned complex medical cases while under the supervision of an attending physician [3]. While some errors may be inevitable, proper training and awareness can help mitigate a trainee’s risk of being named in a lawsuit. Pediatric trainees can still be held accountable and subsequently be included in malpractice suits.

Pediatric patients exhibit distinct characteristics compared to adults, including unique presentation patterns and substantial variations in drug dosing requirements. Prior studies have demonstrated a higher incidence of adverse drug events (ADEs) in pediatric settings compared to adult settings [4]. Consequently, it is imperative for all trainees who encounter the pediatric population to thoroughly review malpractice cases involving pediatric trainees, extracting valuable lessons to avoid repeating similar errors. Despite pediatricians being a small portion of malpractice lawsuits, the impact of such lawsuits on both patients and pediatricians can be severe. Many pediatricians involved in malpractice lawsuits experience physical and psychosocial symptoms such as fatigue, anxiety, and depression [3]. Furthermore, litigation leads to defensive medicine practice, including increased tests and hospitalizations, which are associated with increased healthcare costs [2,3]. Malpractice education and research are therefore essential in every specialty, including pediatrics. Additionally, pediatric patients are among the most vulnerable, requiring specialized knowledge and expertise due to their unique medical needs. Although malpractice lawsuits have been studied in various medical specialties, to our knowledge, no research to date has focused on those involving pediatric trainees [1,5,6]. We hypothesized that we could find common trends in malpractice lawsuits involving pediatric trainees by searching through a legal database. Our objective was to understand the source of pediatric malpractice cases to be able to coach trainees during their practice to best protect themselves.

## Materials and methods

We performed a retrospective study that aimed to identify all medical malpractice cases involving pediatric trainees. The LexisNexis legal database was utilized to search through malpractice cases. LexisNexis is a subscription-based legal research tool, containing all publicly available court records for state and federal jury verdicts and settlements in the United States. LexisNexis is commonly implemented in research and is a leading tool for medical malpractice studies [5,6]. Our search criteria included malpractice cases involving pediatric trainees (interns, residents, and fellows) between January 1, 2000, and December 31, 2021. To identify all potentially relevant medical malpractice cases involving pediatric trainees, we searched using relevant keywords: [(residency or resident or fellow or trainee or “post graduate” or “intern” or “first year” or “1st year” or “second year” or “2nd year” or “third year” or “3rd year” or “fourth year” or “4th year” or fellow or fellowship) and (pediatric*) and (malpractice)]. Our search resulted in 494 cases, and each case was manually reviewed by three authors to only include cases involving pediatric trainees. Cases that met the inclusion criteria were analyzed for key variables, including the allegation, the nature of the injury, date, and the outcome of the case. Cases were included if malpractice occurred following the delivery of a newborn through the care of young adults up to age 21. We only included fellows (for example, pediatric urologists or pediatric surgeons) if the case description specifically stated that the trainees were pediatric fellows. Cases involving prenatal care or care provided during delivery were excluded for significant overlap with trainees within other medical fields. The data analyzed in this study were sourced from public court records. As such, the study was not subject to institutional review board approval according to Title 45 CFR 46.104(d)(4).

## Results

**Figure 1:**
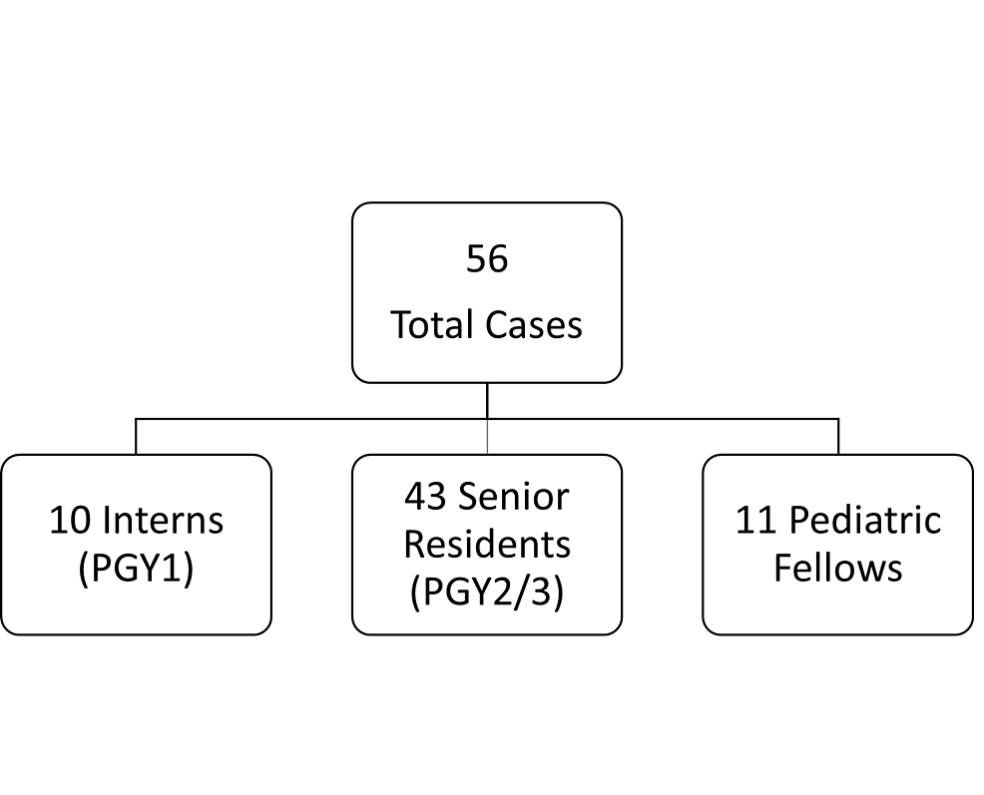
Level of trainees involved PGY, postgraduate year

**Figure 2:**
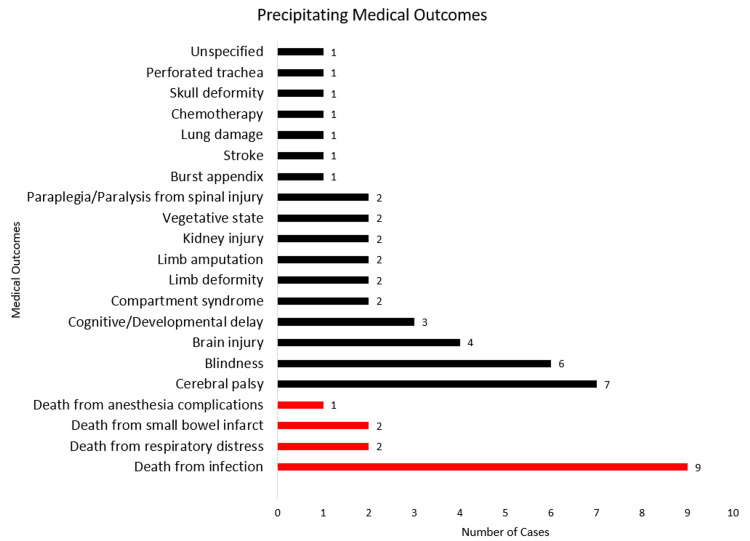
Medical outcomes identified in the cases Red indicates cases that led to death

After a manual review of the 494 cases, 56 of them met our inclusion criteria consisting of 10 pediatric interns, 43 second- or third-year pediatric residents, and 11 pediatric fellows as defendants (Figure 1). A total of nine cases involving surgical procedures performed by pediatric surgical residents or fellows were included. The litigations primarily encompassed instances of death (17), along with a variety of other ailments, such as blindness and neurologic damage (Figure 2). Causes of mortality included sepsis from infection (nine), respiratory distress (two), small bowel infarction (two), and anesthesia complications (one); three cases remained confidential.

**Figure 3:**
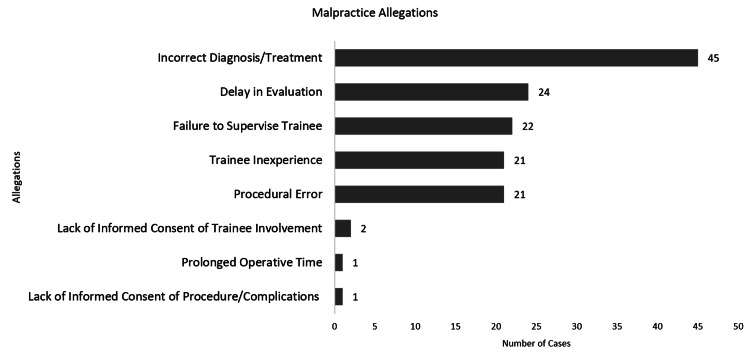
Breakdown of allegations claimed in cases

Incorrect diagnosis or treatment was claimed in 45 (80.3%) cases, delay in evaluation in 24 (42.9%), failure to supervise trainee in 22 (39.3%), trainee inexperience in 21 (37.5%), procedural error in 21 (37.5%), lack of informed consent of resident being involved in two (3.6%), prolonged operative time in one (1.8%), and lack of informed consent of procedure/complications in one (Figure 3).

We identified seven alleged medical errors qualifying as serious reportable events [7]. Serious reportable events, also known as “never events,” often involve serious medical errors, such as wrong-site surgery or medication errors leading to severe harm. ADEs, classified as care management events, were identified in five cases. These errors involved the usage of the wrong drug, improper dose, and improper route of administration. Additionally, we identified two more care management events involving “patient death or serious injury resulting from failure to follow-up or communicate laboratory, pathology, or radiology test results.” The first involved an intern failing to communicate properly with the laboratory to schedule a blood transfusion resulting in patient death; the second involved several communication shortcomings between radiology, orthopedic, and pediatric teams to coordinate the removal of a child’s cast resulting in compartment syndrome. Two cases alleged medical malpractice due to failure to obtain a proper Spanish-speaking medical interpreter. The first of these cases involved the delegation of interpreter duties by a pediatric trainee to an untrained Spanish-speaking social worker, and the second involved complete failure to obtain personnel with any proficiency in Spanish.

**Figure 4:**
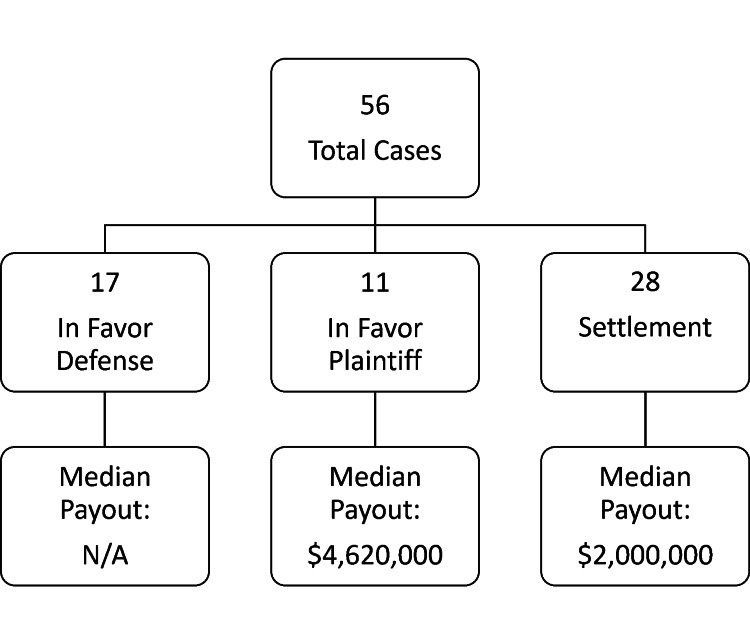
Lawsuit outcomes and payouts

Of the 56 total cases, 28 (50.0%) resulted in settlement, 11 (19.6%) resulted in verdicts favoring the plaintiff, and 17 (30.4%) cases resulted in verdicts in favor of the defense (Figure 4). The average time from medical injury to verdict date was 6.2 years. The median time to verdict in favor of the defense was 6.3 years (interquartile range (IQR), 4.6-7.9 years), the prosecution was 6.8 years (IQR, 5.4-10.3 years), and for settlements was 4.8 years (IQR, 3.4-6.0 years). The damages claimed in settlements averaged $6,090,024 (median, $2,000,000; IQR, $1,562,500-$4,900,000) and in plaintiff verdicts averaged $7,965,760 (median, $4,620,000; IQR, $975,000-$10,100,000). While examining the trend of these cases based on those beginning in the first 10 years versus the last 11 years of inclusion, one of 17 cases with defense verdicts began from 2010 to 2021, zero of 11 cases with plaintiff verdicts, and three of the 29 settlements; these results indicate that most of the lawsuits available on LexisNexis were from the years 2000 to 2009.

## Discussion

Malpractice lawsuits involving pediatric trainees are a reality that can lead to physiologic and psychologic distress in physicians and trainees. Such distress can manifest as frustration, difficulty concentrating, and insomnia, which can further increase the risk of medical error [8-10]. To better understand the common themes and reasons for litigation in pediatric trainees, we conducted a study reviewing legal documents comprising all state and federal malpractice cases involving pediatric trainees. It is worth noting that the majority of malpractice cases did not make it to a higher judicial court and were, therefore, not included in our search. Our study found that the average time from medical injury to verdict was 6.2 years, which is longer than the previously reported average of 5.2 years in malpractice cases from all specialties [11]. On the other hand, our findings on median damages claimed in settlements and plaintiff verdicts are consistent with prior studies, suggesting that the risk of specialty involvement in lawsuits may not necessarily correlate with average payment amounts [1,2]. This is the first study to our knowledge to highlight the details of malpractice lawsuits involving pediatric interns, residents, and fellows. This article was previously posted to the Research Square preprint server on January 5, 2023.

During the 21-year period, lawsuits were much less prevalent during the last 11 years compared to the first 10 years. This is consistent with the National Practitioner Data Bank (NPDB), which similarly reports that medical malpractice payment reports have decreased from 15,395 reports involving MDs or DOs in 2000 to 5,698 in 2021 [12]. While the NPDB reports all paid malpractice claims by trainees and physicians, it is difficult to estimate how many trainees are annually involved in malpractice lawsuits as trainees do not end up making a payment. It is also important to note that trends in malpractice lawsuits can vary over time and may be influenced by factors such as changes in legal regulations, shifts in healthcare practices, variations in public awareness, and evolving attitudes toward litigation [1,2,5,6,13].

In our study of 56 cases (Appendix Table 1), only one specifically mentioned successful litigation against the trainee. Many of the cases did not indicate whether the trainee(s) were included or dismissed from the lawsuit. Most of the cases named the hospital system or attending physician(s) as the primary defendants. Four cases explicitly stated that the trainees were dismissed due to either the judge’s decision or the trainee’s argument for dismissal. For example, in one case, an intern named in a settlement argued that their limited role in the patient’s care and actions ordered by senior residents warranted dismissal from the lawsuit, which was granted. Additionally, two trainees included in a malpractice case asserted that their positions as physicians in training at the time meant that all actions and treatments provided were performed under the direction and supervision of their supervisors. They argued that they performed within the standard of good and proper medical care as performed by residents and were both dismissed from the case prior to its conclusion with a verdict in favor of the defense. These findings differ from the 1991 Centman v. Cobb ruling, which stated that interns or first-year residents are “practitioners of medicine required to exercise the standard of care applied to physicians with unlimited licenses to practice” [13].

The most common allegations in our study were diagnostic or treatment errors, delay in evaluation, failure to supervise trainees, trainee inexperience, and procedural error. These findings are consistent with a previous study on United States malpractice cases, which found that diagnostic error was the leading allegation, with death and significant permanent injury as the leading injuries from these claims [14]. In our study, seven cases were due to ADEs. Previous studies have cited ADEs as the most prevalent of the serious reportable events, with higher rates reported in pediatric settings compared to adult settings [4]. The need for weight-based dosing and diluting stock solutions in pediatric patients have been identified as factors contributing to an increased occurrence of malpractice lawsuits [4]. An alarming finding from our study was the existence of two cases alleging medical malpractice due to the failure to obtain a proper Spanish-speaking medical interpreter. Previous research has confirmed that medical personnel receive insufficient training in working with interpreters [15-17].

Many medical errors are preventable. It is important for physician trainees at all levels, from intern year through fellowship, to receive training on common mistakes that can lead to potential lawsuits and how to best protect themselves if an error occurs. Diagnostic and treatment error remains the most common across all specialties and can be reduced through strong communication among the healthcare team, patient, and family. Trainees should also follow the process of diagnostic modification and refinement, starting with a broad differential and refining as more information is gathered through clinical assessment and laboratory findings [18,19]. Closing the gap between trainees and leadership is also important. Attending physicians should be available to assist and advise trainees when needed, and trainees should feel confident in reporting errors to their supervisors, who should provide support [20]. Finally, the proliferation of technology and online resources has greatly expanded the availability of both strong, evidence-based medicine and weaker, non-evidence-based medicine. Trainees must be able to distinguish between medical sources and only use clinical sources supported by established guidelines [4].

There are several limitations to our study. First, cases that were not tried in state or federal court, or that were settled outside of court, handled by a third-party arbitration such as an insurance company, or appealed at the district level were not included because LexisNexis contains only cases filed at the state or federal level [21]. Therefore, this sample of malpractice cases involving trainees is not representative of all such cases. Second, the cases accessible on LexisNexis are based on court documents written by judges and lawyers, without access to medical records, which limits the ability to corroborate the information reported by plaintiffs and defendants [21]. Third, cases that may have involved pediatric trainees but did not explicitly state this detail or involvement were excluded from our findings. Fourth, trainees can and are often dropped at any point in the litigation due to financial factors or other reasons, but details about this were unclear in the examined cases. Despite these limitations, the information provided in these cases presents a learning opportunity for trainees and residency programs. Future research should delve into a broader range of specialties to provide a more comprehensive understanding of malpractice incidents involving trainees across the spectrum of medical practice, enabling the development of more effective training programs and patient safety measures.

## Conclusions

Malpractice cases involving pediatric trainees, akin to their counterparts in other disciplines, are an undeniable occurrence within the realm of medical practice. Pediatric patients demonstrate notable dissimilarities when compared to adults, underscoring the importance for trainees who encounter this population to diligently examine malpractice cases involving fellow pediatric trainees. By extracting invaluable lessons from these cases, trainees can prevent the recurrence of similar errors. Given the interplay of supervision and diagnostic accuracy, trainee education and faculty development should emphasize malpractice education and strategies to mitigate lawsuits to both improve patient outcomes and reduce the likelihood of future malpractice claims.

## Appendices

**Table 1:** Malpractice case descriptions PICU, pediatric intensive care unit; ED, emergency department 1, delay in evaluation; 2, incorrect diagnosis/treatment; 3, procedural error; 4, lack of informed consent of procedure/complication; 5, lack of informed consent of the trainee being involved; 6, failure to supervise a resident; 7, the trainee was inexperienced; 8, prolonged operative time

Supplemental Table 1: Case summaries						
State	Days from incident to verdict	Plaintiff award in USD	Allegations	Precipitating medical issue	Chief allegation	Case summary
Outcome: in favor of the plaintiff						
WA	1612	15,255,971	2,7	Neurologic damage	Incorrect treatment	A fellow provided improper medication instructions to the mother of a child post-heart transplant who developed an upper respiratory infection. The child was given a specifically contraindicated medication and suffered from cardiac arrest and hypoxic brain injury.
OH	Unknown	6,500,000	2	Death: myocarditis	Incorrect diagnosis	A child diagnosed with apparent heart failure after presenting to ED was transported for evaluation by a pediatric intensivist fellow and resident. Treatment was initiated for hypovolemic shock without evaluation of the heart. Shortly after, the child died from heart failure due to viral myocarditis.
PA	Unknown	1,200,000	1,2,7	Death: unknown	Trainee inexperience	A child admitted to the pediatric nephrology floor died. The allegations stated interns or residents were inexperienced, and the child's condition worsened because the defendants did not act on warning signs.
MO	3211	10,200,000	1,2	Neurologic damage	Delay in evaluation	A newborn developed signs of respiratory distress hours after birth. Plaintiffs alleged that the third-year pediatric resident failed to appropriately monitor the baby within the first few hours of life, and as a result, he suffered hypoxic ischemia causing permanent brain injury and cerebral palsy.
LA	5434	34,097,393	2	Lung and neurologic damage	Incorrect treatment	The chief pediatric resident failed to provide proper postnatal care to a critically ill newborn. Allegations stated the resident failed to immediately initiate treatment leading to irreversible brain damage.
RI	Unknown	400,000	2	Death: RSV	Incorrect treatment	A first-year resident helped triage an ill infant brought to the ED by a Spanish-speaking family. No interpreter was utilized for gathering information from the family, and the infant was subsequently discharged. He died several hours later due to RSV.
LA	6308	10,000,000	3,7	Compartment syndrome	Trainee inexperience	A resident at a children's hospital improperly set a toddler's fractured arm in a cast, leading to compartment syndrome and permanent loss of use of the arm.
PA	2027	500,000	3,6	Deformity/discomfort	Failure to supervise a resident	An unsupervised pediatric resident performed circumcision on a newborn, resulting in deformity of the genitalia.
NY	1765	4,620,000	2	Blindness	Trainee inexperience	A pediatric resident failed to properly communicate with radiology regarding a child's MRI. The child was discharged with a large intracranial blood clot and ultimately suffered blindness.
VA	2019	750,000	3,5,6,7	Blindness	Failure to supervise a resident	Two residents performed a procedure unsupervised by an attending physician, resulting in blindness. The child's parents were not informed of resident involvement.
OH	2997	4,100,000	1,2,6	Leg amputation	Trainee inexperience	A resident failed to recognize signs of thromboembolism in a child's leg, resulting in delayed treatment and eventual amputation.
Outcome: settlement						
MA	Unknown	1,600,000	1,2,6,7	Death: pericardial effusion	Delay in evaluation	A pediatric intern and resident failed to evaluate a child in a timely manner. Incorrect diagnosis and treatment of the child's condition resulted in rapid deterioration and eventually death.
NY	Unknown	3,625,000	2,3	Sustained complications of kidney transplant	Incorrect treatment	Defendants, including one pediatric resident, provided incorrect treatment for a child with end-stage renal disease. The child sustained lifelong complications.
Unknown	2253	2,400,000	1,2,3,6,7,8	Loss of a portion of the bowel	Trainee inexperience	A pediatric resident evaluating a child with severe abdominal pain failed to recognize signs of pancreatitis. The defendant also failed to notify a supervising attending physician, resulting in delayed treatment and severe complications from subsequent septic shock.
MA	Unknown	3,795,820	2,6	Blindness	Incorrect diagnosis	A pediatric neurology fellow, supervised by a neurosurgeon, failed to recognize signs exhibited by a plaintiff of severely increased intracranial pressure, leading to severe cortical visual impairment.
VA	555	1,550,000	1,2,6,7	Death: meningitis	Incorrect diagnosis	A third-year pediatric resident failed to order proper diagnostic studies for an infant with suspected meningitis. The supervising attending was informed of the situation and failed to obtain the studies as well.
MI	Unknown	1,995,000	1,2	Neurologic damage	Incorrect treatment	A pediatric resident and neonatal fellow failed to provide proper treatment to a newborn with suspected meningitis in the NICU. The newborn was transferred back to the mother's room and suffered rapid deterioration with lifelong complications.
HI	Unknown	11,000,000	2,3,6,7	Cerebral palsy	Procedural error	A newborn with respiratory distress was improperly intubated by a first-year pediatric resident. The supervising second-year resident failed to identify the error, resulting in severe hypoxic neurologic damage.
MA	Unknown	Unknown	2	Deformity and seizures	Incorrect treatment	A pediatric resident evaluating a child in ED fails to correctly identify and treat complicated sinusitis. Delayed treatment caused progression to osteomyelitis, ultimately necessitating craniotomy.
TX	1205	Unknown	2,3	Death: cerebellitis	Incorrect diagnosis	A pediatric fellow was involved in the failure to identify structural changes in a child's brain on imaging. No treatment was provided to the child resulting in death from cerebellitis.
PA	Unknown	1,200,000	2,3	Death: respiratory distress	Incorrect treatment	A pediatric resident and two interns involved in caring for an infant with respiratory distress failed to identify significant hypoxia, resulting in death.
NY	2614	3,000,000	1,2,7	Adverse medication effects	Incorrect diagnosis	A pediatric trainee was involved in the failure to correctly diagnose a child with aggressive metastatic cancer. The delay in treatment required more aggressive chemotherapy with several long-term adverse effects for the child.
NY	2150	3,990,000	2,3	Neurologic damage	Procedural error	A neonatal fellow caring for ill newborns improperly administered medications, resulting in severe hypoglycemia with permanent neurologic damage.
NY	1806	1,750,000	3,5,6,7	Neurologic damage	Failure to supervise trainee	A pediatric resident and intern performed a spinal tap on an infant who became unresponsive. A plaintiff argues that trainees were inadequately supervised and displayed negligence while observing breathing and responsiveness. The infant suffered an ischemic brain injury and chronic seizure disorder.
NY	1676	675,000	1,2	Compartment syndrome	Incorrect diagnosis	Pediatric residents contributed to incorrect diagnosis and treatment of postoperative compartment syndrome. Delayed treatment resulted in several complications, including permanent nerve damage.
CA	1301	5,000,000	3,6	Neurologic damage	Trainee inexperience	A pediatric intern notified of a newborn's respiratory distress failed to notify the attending physician and initiated improper treatment, leading to cardiopulmonary arrest. Plaintiffs claimed that the care team failed to meet the proper standard of care.
CA	1365	6,674,318	1,2,3,7	Neurologic damage	Incorrect diagnosis	A pediatric resident was involved in the failure to detect spinal arteriovenous malformation, resulting in paraplegia. The child was misdiagnosed with Guillain-Barre syndrome.
Unknown	Unknown	Unknown	1,3	Neurologic damage	Delay in evaluation	Pediatric residents failed to properly monitor an infant following central line placement. The child's condition deteriorated, and an attempt at intubation failed, resulting in cardiopulmonary arrest with permanent neurologic damage.
NY	1545	70,000,000	1,2,3,6,7	Neurologic damage	Incorrect treatment	A second-year resident was monitoring a premature infant who suddenly desaturated. The resident incorrectly sought treatment for assumed pneumothorax and proceeded to attempt needle aspiration four times. The baby suffered a very complicated course after the incident with permanent hypoxic brain damage.
IL	Unknown	6,000,000	1,2,3,6,7	Neurologic damage	Procedural error	Residents failed to detect or replace a dislodged tracheal tube while treating an infant's labored breathing, resulting in a vegetative state due to oxygen deprivation.
NY	1314	2,000,000	1,2,3,6,7	Deformity	Failure to supervise trainee	A pediatric resident was involved in improper treatment of child's compartment syndrome, resulting in permanent leg deformity. The resident allegedly performed traction unsupervised while referring to a medical textbook.
NY	2855	2,000,000	2,6,7	Kidney damage	Incorrect treatment	A pediatric intern working in PICU mistakenly ordered 45 times the proper dose of potassium for an infant. The infant received the incorrect dose and suffered permanent renal damage, eventually requiring a transplant.
FL	957	75,397.43	2,3	Loss of mobility	Delay in evaluation	A child receiving post-operative care from a team, including two pediatric trainees, was improperly monitored. The child developed complications requiring 13 additional surgeries with permanent loss of mobility.
NY	2890	850,000	3,6,7	Neurologic damage	Failure to supervise trainee	A pediatric resident resuscitating a newborn departed from the standard procedure by failing intubation twice. The infant was not stabilized until an anesthesiologist arrived, and the attending neonatologist was deemed negligent in leaving the trainee unsupervised. The infant experienced seizures and permanent developmental and cognitive deficits.
PA	Unknown	Unknown	3	Perforated trachea	Procedural error	Improper intubation of a newborn baby performed by a resident resulted in a perforated trachea.
VA	1073	200,000	2,6,7	Death: septic shock	Failure to supervise trainee	A plaintiff argued that a children's hospital had improper procedures to adequately supervise or train its residents after inadequate follow-up resulted in a child's death from septic shock. The child had been discharged earlier that day following skin grafting to treat severe burns. When the mom called to report a new fever, she was informed by a trainee to only give the child Motrin without consideration for the child's recent hospitalization.
NJ	994	4,600,000	1	Blindness	Delay in evaluation	A premature infant, who received oxygen therapy, did not receive a standard ophthalmologic exam within the proper timeframe, resulting in blindness. A plaintiff sued a neonatologist, the chair of the neonatology department, and a pediatric resident.
AL	1632	Unknown	1,2,3,6	Arm amputation	Incorrect treatment	An unsupervised pediatric trainee caring for a child with arterial trauma after attempted IV placement forms an improper treatment plan. The delay in appropriate treatment necessitated arm amputation.
TX	1460	Unknown	1,2,6,7	Neurologic damage	Incorrect treatment	A child presented to the children's ER with septic meningitis and was attended to by nurses, pediatric residents, and medical students. Treatment was not provided for six hours, and the child developed seizures, resulting in cardiopulmonary failure.
Outcome: in favor of the defense						
OH	3572	None	6,7	Neurologic damage	Trainee inexperience	A resuscitation team, including a pediatric intern and second-year and third-year residents, allegedly delayed timely resuscitation for a critically ill infant by failing intubation attempts and failing to summon a fellow.
NY	2800	None	1,2,7	Death: anesthesia complications	Delay in evaluation	A third-year resident was involved in evaluating a child with suspected septic shock. The child passed away from anesthesia complications, and a plaintiff argued that delay in evaluation was the primary cause.
FL	2664	None	1,2,6	Death: hypoxia	Delay in evaluation	A plaintiff argued that first-year residents failed to timely order a blood transfusion for a child with critically low hemoglobin levels, leading to cardiac arrest and death.
GA	3774	None	2,3	Neurologic damage	Procedural error	An infant sustained a period of inadequate oxygenation due to improper endotracheal tube placement under the care of a neonatology fellow and attending physicians. After appealing on the basis of entitlement to official immunity, a fellow was dismissed.
CA	879	None	4	Neurologic damage	Lack of informed consent for procedure/complication	A pediatric fellow was involved in a suit claiming lack of informed consent by a Spanish-speaking family. The fellow allegedly utilized a Spanish-speaking social worker who allegedly did not fully explain the complications of the procedure. The parents stated that they explicitly did not give consent for the procedure, which was still performed.
CA	1015	None	2	Death: congenital gut malrotation	Incorrect diagnosis	A pediatric attending and fellow allegedly missed the diagnosis of gut malrotation in a child who died of surgically preventable complications.
NY	4529	None	1,2,6	Blindness	Incorrect diagnosis	Several pediatric residents were involved in allegations of failure to diagnose congenital glaucoma and delay in referral to ophthalmology.
NY	1842	None	1,2	Death: meningitis	Delay in evaluation	A child with meningitis died after the initiation of antibiotic treatment was delayed. Plaintiffs included several unspecific pediatric residents with involvement in the child's evaluation and treatment.
OH	Unknown	None	2	Appendicitis complications	Incorrect diagnosis	A pediatric resident misdiagnosed appendicitis as a urinary tract infection; the appendix burst three days later.
FL	1848	None	2	Neurologic damage	Incorrect treatment	A second-year resident allegedly discharged a child with instructions to follow up with a cardiologist within the next week. The patient did not and suffered an ischemic stroke caused by an issue with his mechanical heart valve.
MA	2627	None	1,2	Death: unknown respiratory distress	Delay in evaluation	A parent of a child with respiratory distress utilized a hospital answering service to seek medical advice. They argued that the resident failed to return calls, and when they called the next day, they informed the parent that the child's symptoms did not warrant treatment. The resident argued that they called three times without receiving an answer and instructed the parent to bring the child to the hospital for evaluation if symptoms continued. The child died from respiratory distress.
NY	1313	None	2	Death: myocarditis	Incorrect treatment	A child with heart failure and myocarditis died under the care of a senior pediatric resident and attending physician.
PA	1788	None	1,2,6,7	Death: sepsis	Trainee inexperience	A pediatric resident was involved in alleged negligent care of a critically ill newborn with the failure to call an attending pediatrician. A newborn died from *Escherichia coli* sepsis.
NY	3105	None	2	Temporary blindness	Delay in evaluation	Plaintiffs argued that the defendants failed to timely diagnose and treat a child's rare condition causing pain and temporary loss of vision. A pediatric resident was granted dismissal after asserting that they were not liable for the actions of attending physicians and didn't deviate from accepted medical practice.
PA	1999	None	1,2	Death: small intestine infarction	Trainee inexperience	Parents of an infant who died from complications of Hirschsrung's disease claimed that a pediatric resident failed to obtain the infant's full medical history and called for evaluation by a surgeon. The defendant argued that the parents were not compliant with answering questions, resulting in failure to document the pertinent history.
NY	4190	None	2	Not specified	Procedural error	A resident in the neonatology department was included in a malpractice claim, alleging deviation from the standard of care and causing injury while assisting an attending neonatologist with the care of preterm twins.
PA	1039	None	2	Death	Incorrect treatment	Medication ordered by a resident working in PICU was not provided to a child, resulting in cardiac arrest.
